# N addition alters growth, non-structural carbohydrates, and C:N:P stoichiometry of *Reaumuria* *soongorica* seedlings in Northwest China

**DOI:** 10.1038/s41598-022-19280-8

**Published:** 2022-09-13

**Authors:** Tingting Xie, Lishan Shan, Wanting Zhang

**Affiliations:** 1 College of Forestry, Gansu Agricultural University, Lanzhou, China; 2 Zhangye Academy of Forestry, Zhangye, China

**Keywords:** Ecology, Physiology

## Abstract

*Reaumuria soongorica* is an important biological barrier for ecological protection in the Gobi Desert in northwestern China, where soil nitrogen availability is low. N deposition has recently increased significantly in Gobi Desert, and the responses of *R. soongorica* to N enrichment may become a problem for ecological restoration and protection. However, little is known about the effects of N addition on the biomass, non-structural carbohydrates (NSC), and carbon:nitrogen:phosphorus (C:N:P) stoichiometry of *R. soongorica* in this region. Here, we examined changes in biomass, NSC and C:N:P ratios of different organs of *R. soongorica* seedlings in four N addition treatments: 0 (N_0_), 4.6 (N_1_), 9.2 (N_2_), and 13.8 (N_3_) g m^−2^ year^−1^. N addition up to 9.2 g m^−2^ year^−1^ significantly increased the biomass of different organs, simultaneously increasing the belowground: aboveground ratio of *R. soongorica* seedlings. Root NSC concentrations significantly increased under all N addition treatments, but leaf and stem NSC concentrations only increased under the N_1_ and N_2_ addition treatments. Nitrogen addition enhanced the soluble sugar concentrations (SSC) of leaves and roots, and reduced starch concentrations (SC) of all organs. Stem and root N concentrations significantly increased under the N_2_ and N_3_ treatments, and leaf N concentrations only increased under the N_3_ treatment, but N addition had no significant effect on plant C and P concentrations. Leaf and stem C:N ratios decreased significantly under the N_2_ and N_3_ treatments, but root C:N decreased significantly in all N addition treatments. The N_3_ treatment significantly increased the N:P ratio of all organs. N addition significantly enhanced available N (AN), available P (AP) and total phosphorus (TP) in rhizosphere soil. Our results suggest that N addition alters the biomass, NSC, N concentrations, C:N and N:P ratios of all plant organs, but roots responded more strongly than stems or leaves to N addition, potentially allowing the plants to absorb more water from the arid soil in this region ensuring the survival of *R. soongorica* seedlings. Rhizosphere soil AP, AN and TP concentrations were important factors affecting the NSC concentrations and stoichiometric characteristics of *R. soongorica*.

## Introduction

Since the industrial revolution, atmospheric nitrogen (N) deposition has increased rapidly due to the widespread use of N-containing synthetic fertilizers and increased fossil fuel combustion, motivating increased research on the influence of N deposition on terrestrial ecosystems^1,2^. It has been shown that N deposition can affect plant photosynthesis and nutrient transport^3^, and accordingly the form and distribution of carbohydrates, the main product of photosynthesis, in plant organs should be affected by N deposition^4^. N deposition has also been shown to affect the stoichiometry (carbon, nitrogen, and phosphorus) of plants in terrestrial ecosystems and thus alter plant physiological activity and growth rates^5^.

Non-structural carbohydrates (NSC) include soluble sugars and starch. These NSCs provide the main energy supply for plant growth, development and reproduction, and perform important functions in metabolism, transporting water and assimilation products, regulating osmotic potential, and acting as a nutrient reservoir^6,7^. Understanding plant NSC concentrations and their variation among organs is thus the ideal entry point to explore the response and adaptation of plant growth and physiology to environmental stressors. Under environmental stress, plants will alter C allocation among different organs. For example, drought either significantly increased or maintained total NSC concentrations in aboveground plant organs, but reduced total NSC concentrations in sapling roots^8^. N and P fertilization greatly reduced leaf, stem, and root NSC concentrations in *Moringa oleifera* seedlings, possibly because of the dilution effects of increased biomass following fertilization^9^. Thus NSCs and their components (soluble sugar and starch) display different responses to different environmental factors among different organs^10,11^. Understanding fluctuations in NSC concentrations under different types of environmental stress has become an important tool for exploring plant adaptation strategies to diverse stressors.

Carbon (C), nitrogen (N) and phosphorus (P) are structural elements and the major nutrients maintaining biogeochemical cycling and energy flow within ecosystems^12^. Many studies have found that N addition significantly increased the C, N and P contents of plant organs^13–15^. Plant C:N and C:P ratios usually increased^16^ or decreased^17^ under N addition, but plant N:P ratios may increase^15^, decrease^18^ or remain the same^17^ depending on the species^18^ and soil nutrient conditions^19^ of the study. However, most studies have focused on aboveground stoichiometry rather than whole-plant stoichiometry^20,21^, and the responses of different plant organs to N addition remain largely unexplored in the Gobi Desert region.

Under natural conditions, *Reaumuria soongorica* can reproduce through seeds. The success of the plant’s natural renewal mainly depends on seed germination and seedling survival. In the Gobi Desert region, nitrogen is a key factor restricting vegetation growth, distribution, and the restoration of damaged ecosystems. The germination of *R. soongorica* seeds and the survival of seedlings are thus likely affected by nitrogen availability. N deposition may be beneficial for *R. soongorica* growth, but recent studies on *R. soongorica* have focused on growth^22,23^, photosynthetic physiology^24,25^, and genetics^26^ in recent years, and little is known regarding the impacts of N on the biomass, C:N:P stoichiometry, and NSC distribution among organs of *R. soongorica* seedlings in the Gobi Desert in northwestern China. To evaluate the effects of nitrogen on C:N:P stoichiometry and NSC content in different organs of *R. soongorica*, we conducted an N addition experiment. We hypothesized that (1) N addition would significantly enhance the biomass of different organs, but aboveground (leaf and stem) biomass would respond more strongly than belowground (root) biomass, resulting in a lower belowground: aboveground biomass ratio; (2) N addition would significantly enhance the NSC content of different organs, and (3) N addition would significantly enhance N concentrations in plant organs and soil, resulting in lower plant C:N ratios and higher N:P ratios. Our study is important for revealing the responses and adaptation mechanisms of desert plants to N deposition.

## Results

### Biomass across organs

**Figure 1:**
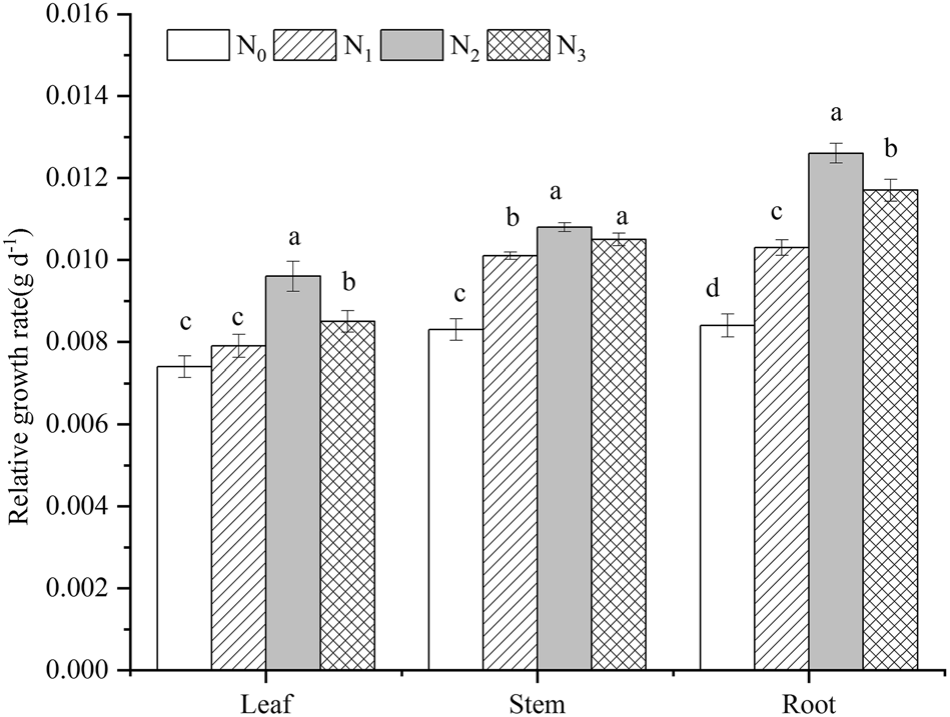
Effects of N addition on the relative growth rate of *R. soongorica*. Error bars represent SE (n = 6). Different lowercase letters above bars indicate significant differences at *P* < 0.05.

With increasing N addition, total, leaf, stem, and root biomass all increased significantly and reached their maximum values in the N_2_ treatment (*P* < 0.05). Compared with the N_0_ treatment, total, leaf, stem and root biomass increased by 60.49%, 42.85%, 48.86% and 97.5%, respectively, in the N_2_ addition treatment. The ratio of belowground to aboveground biomass also significantly increased in the N_2_ and N_3_ treatments (*P* < 0.05) (Table 1). The relative growth rates (RGR) of leaves, stems and roots were all highest in the N_2_ treatment; stem and root RGRs differed significantly from the control in the N_1_–N_3_ addition treatments (Fig. 1), while leaf RGR was significantly enhanced in the N_2_ and N_3_ treatments compared to the control (*P* < 0.05). Among the different organs, the RGR of roots was highest under N addition, demonstrating that root biomass responded more rapidly than did leaf and stem biomass.

**Table 1:** Effects of N addition on biomass of *R. soongorica.*

N treatments	Total (g)	Aboveground		Belowground	Belowground to aboveground ratio
		Leaf biomass (g)	Stem biomass (g)	Root biomass (g)	
N0	2.86 ± 0.10d	1.19 ± 0.01c	0.88 ± 0.05c	0.8 ± 0.06d	0.39 ± 0.02c
N1	3.53 ± 0.08c	1.30 ± 0.09bc	1.18 ± 0.03b	1.08 ± 0.06c	0.44 ± 0.02bc
N2	4.59 ± 0.04a	1.70 ± 0.08a	1.31 ± 0.04a	1.58 ± 0.05a	0.53 ± 0.03a
N3	4.05 ± 0.11b	1.42 ± 0.13b	1.26 ± 0.05ab	1.36 ± 0.07b	0.51 ± 0.03ab

The data was mean ± SE. Different lowercase letters in each column indicate significant differences among N addition at *P* = 0.05.

### NSC concentrations in different organs

**Figure 2:**
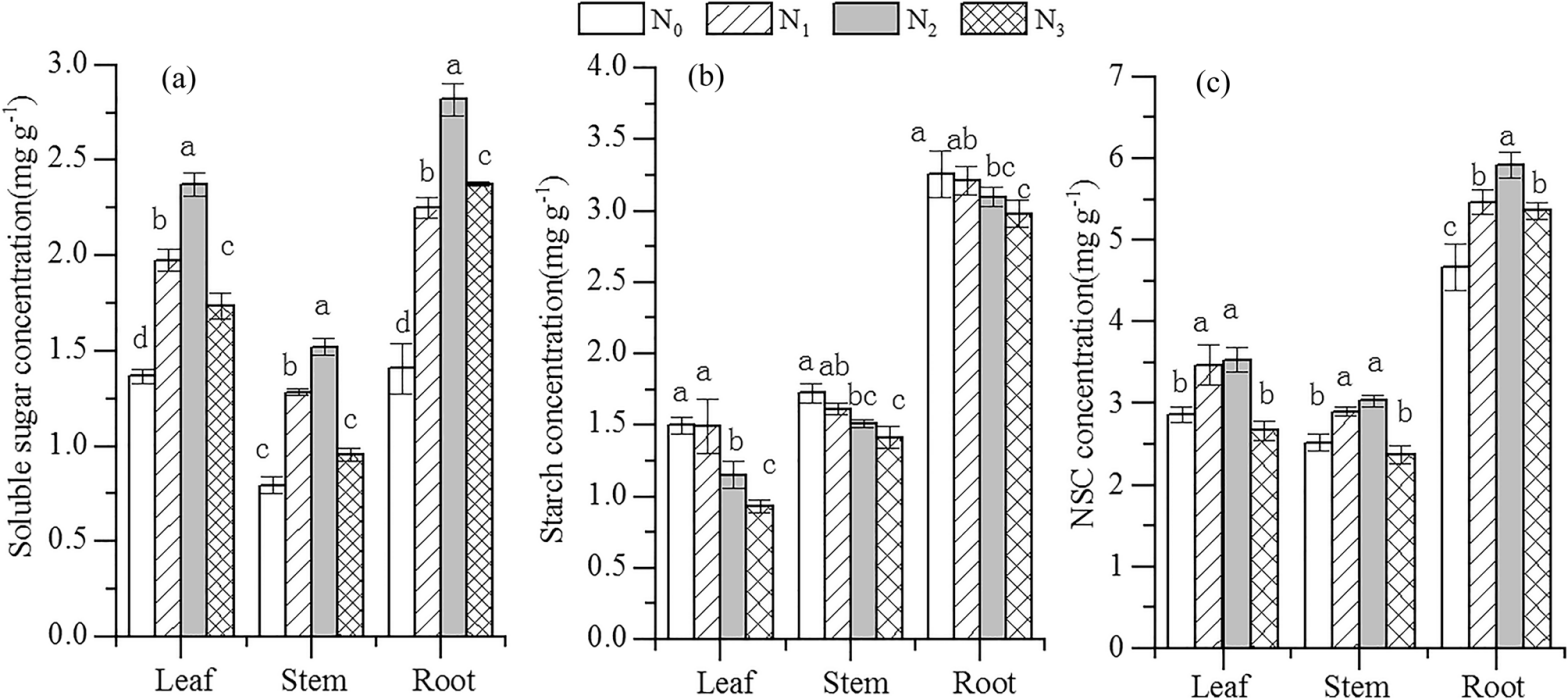
Effects of N addition on soluble sugar, starch, and NSC concentrations of *R. soongorica*. Error bars represent SE (n = 6). Different lowercase letters above bars indicate significant differences at *P* < 0.05.

The soluble sugar concentrations (SSC), starch concentrations (SC) and NSC concentrations of different organs altered with increasing N addition. With increasing N addition, leaf, stem, and root SSC all increased significantly in the N_1_ and N_2_ addition treatments, but decreased significantly (by 26.95%, 37%, and 15.68% in leaves, stems, and roots) in the N_3_ treatment compared to the N_2_ treatment (Fig. 2a) (*P* < 0.05). Leaf, stem, and root SC decreased significantly in the N_2_ and N_3_ addition treatments compared to the control (Fig. 2b) (*P* < 0.05). Compared with N_0_, N addition (N_1_–N_3_) significantly enhanced the NSC concentrations of roots, which were highest in the N_2_ treatment, but leaf and stem NSC concentrations only increased significantly in the N_1_ and N_2_ treatments (Fig. 2c) (*P* < 0.05).

### Nutrient concentrations and stoichiometry across organs

**Figure 3:**
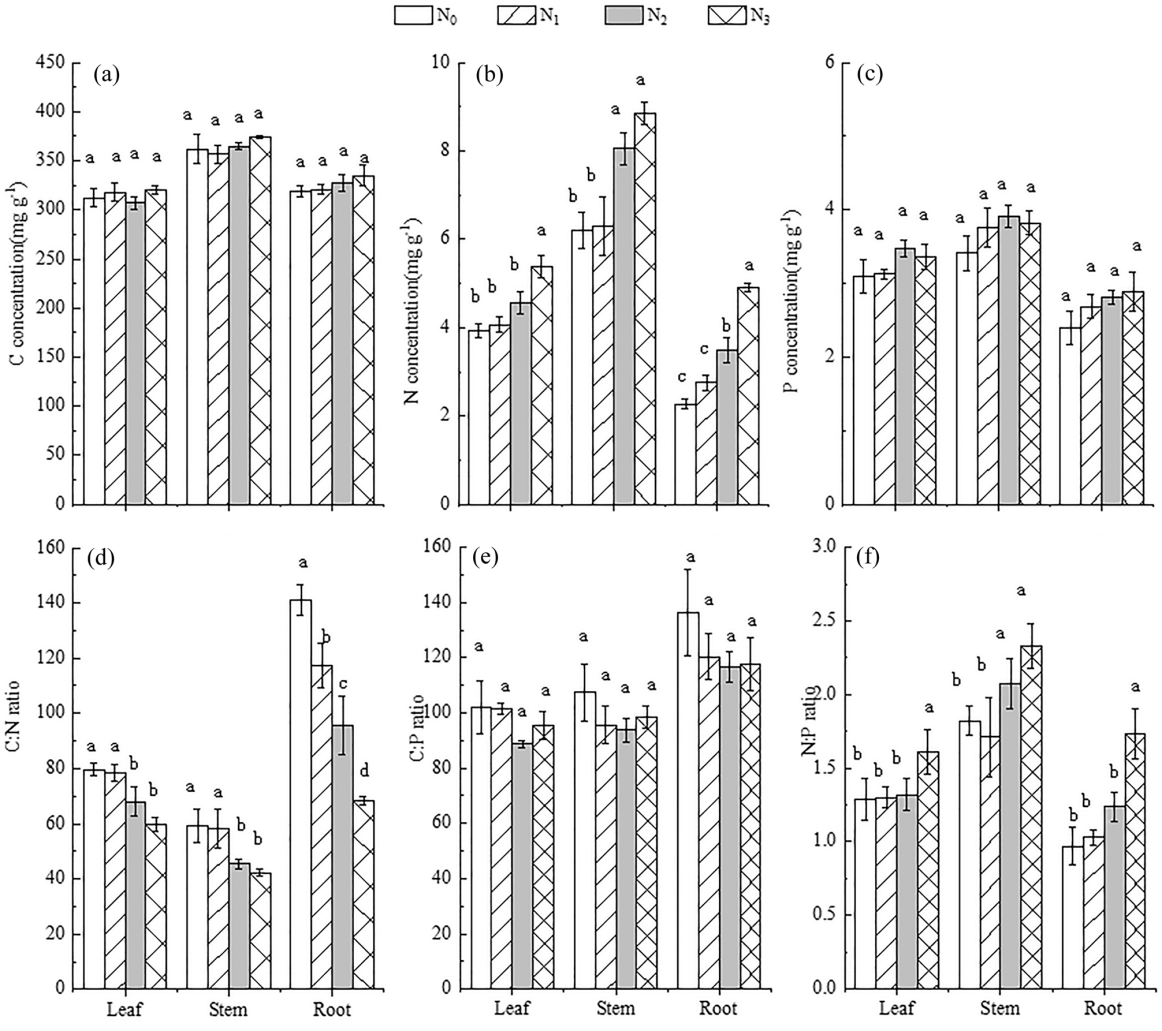
Effects of N addition on plant biomass, C, N, and P concentrations, and the stoichiometric ratios of C:N, C:P, and N:P in *R. soongorica*. Error bars represent SE (n = 6). Different lowercase letters above bars indicate significant differences at *P* < 0.05.

N addition had no effect on P concentrations across all organs (Fig. 3a,c). Compared with N_0_, the N concentrations of stems and roots significantly increased in the N_2_ and N_3_ treatments; leaf N concentration was significantly enhanced in the N_3_ treatment (*P* < 0.05). There were no significant differences in N concentrations between the N_0_ and N_1_ treatments (Fig. 3b).

Leaf and stem C:N ratios decreased significantly in the N_2_ and N_3_ treatments compared to the control, but root C:N decreased significantly with increasing N addition (*P* < 0.05) (Fig. 3d). Compared with N_0_, the C:P ratio of different organs all reduced slightly with N addition, but displayed no significant differences among N treatments (*P* > 0.05) (Fig. 3e). The higher N addition treatments (N_2_–N_3_) significantly enhanced the stem N:P ratio, but leaf and root N:P ratios only increased in the N_3_ addition treatment (Fig. 3f) (*P* < 0.05).

### Rhizosphere soil nutrient concentrations

**Figure 4:**
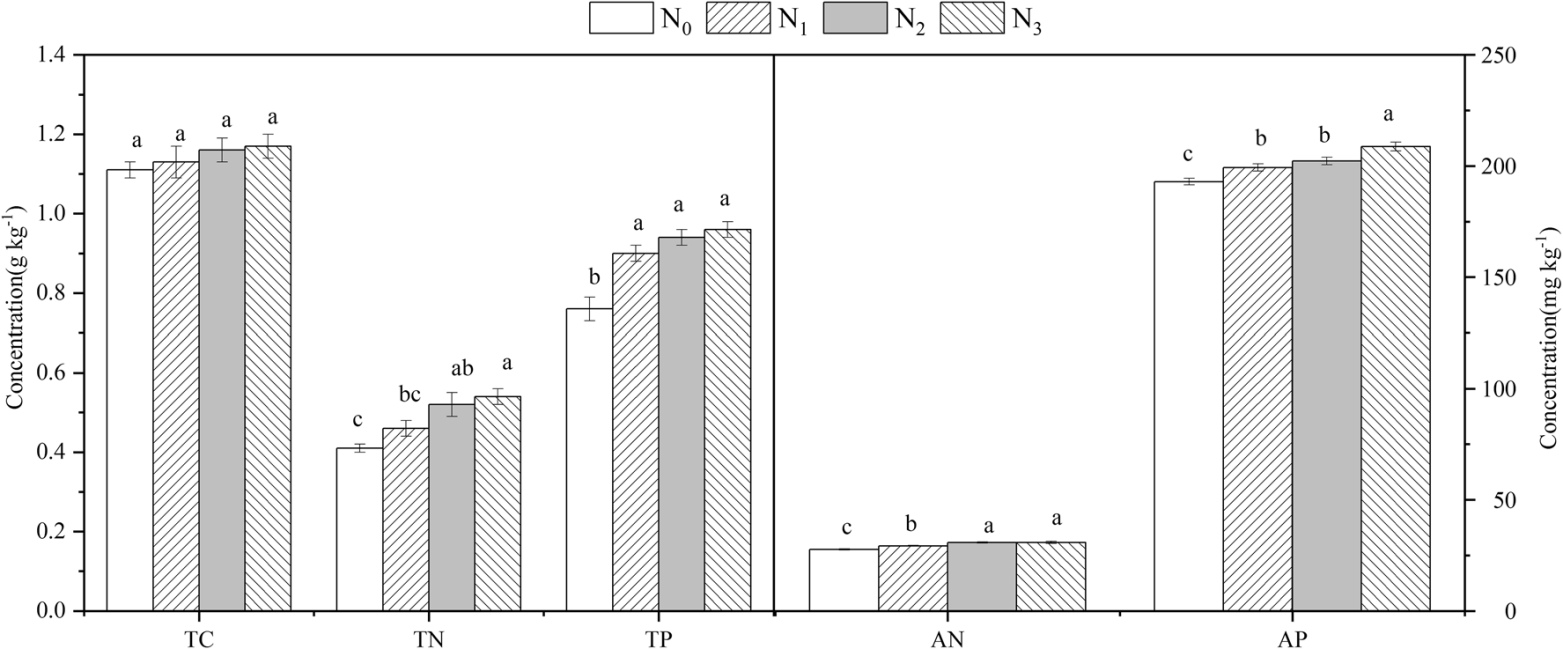
Effects of N addition on soil nutrient concentrations of *R. soongorica*. Error bars represent SE (n = 6). Different letters above bars indicate significant differences at *P* < 0.05.

Compared with N_0_, N addition significantly increased all rhizosphere soil nutrient concentrations [except total carbon (TC) and total nitrogen (TN) in the N_1_ treatment] relative to the control (*P* < 0.05). Rhizosphere soil total phosphorus (TP) did not differ significantly among the N_1_–N_3_ treatments (*P* > 0.05) (Fig. 4a). Available N (AN) was significantly higher in the N_2_ and N_3_ treatments than in the N_1_ addition treatment, but available P (AP) in the N_3_ addition treatment was significantly higher than in the N_1_ and N_2_ addition treatments (*P* < 0.05) (Fig. 4b).

### Relationship between measured soil factors, plant stoichiometric characteristics, and NSC

**Figure 5:**
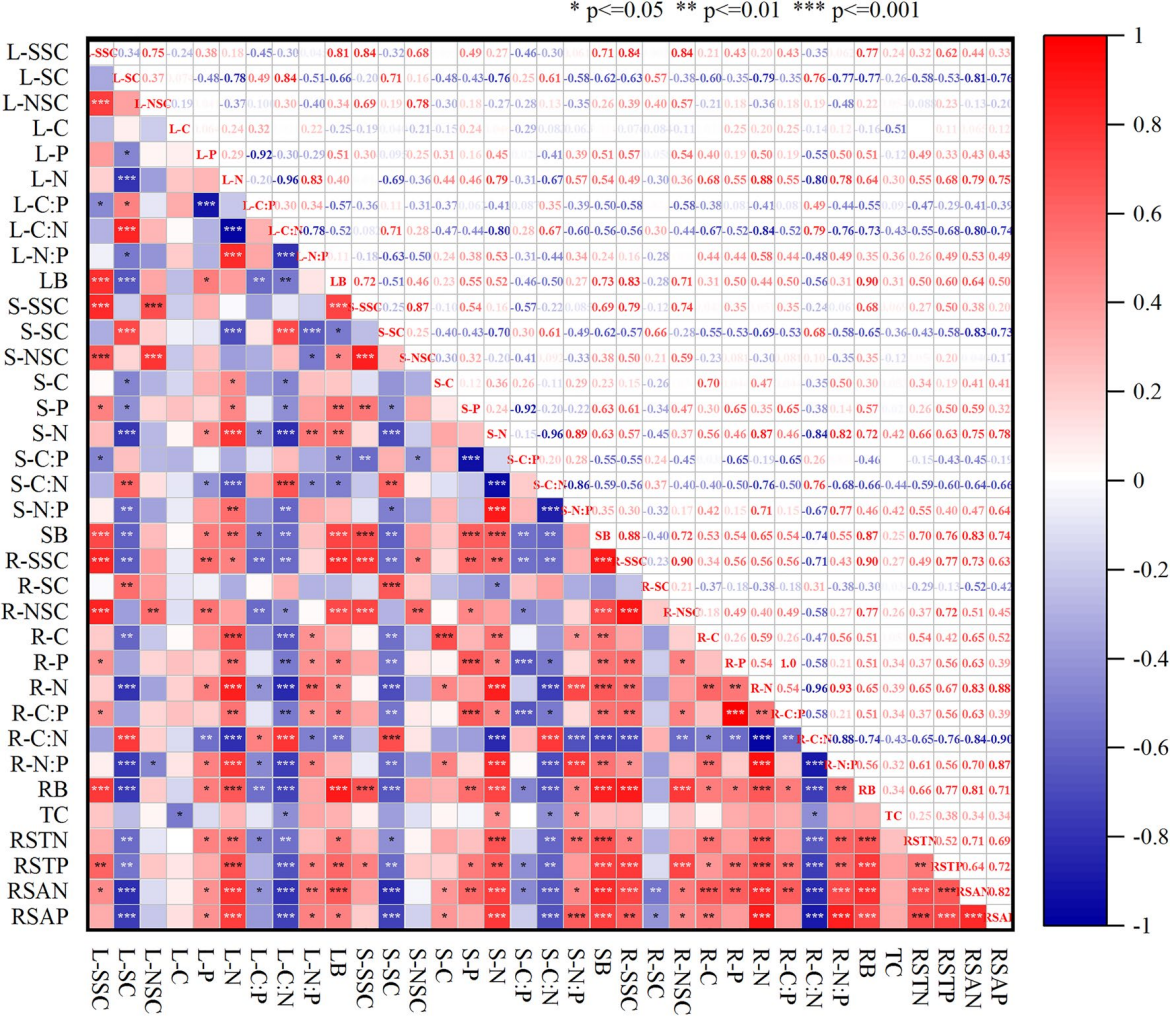
Correlation analysis between soil factors and ecological stoichiometry and NSCs of *R. soongorica*. RSAP = rhizosphere soil available P, TC = rhizosphere soil total carbon, RSTP = rhizosphere soil total phosphorus, RSTN = rhizosphere soil total nitrogen, RSAN = rhizosphere soil available N, L-SSC = leaf soluble sugar concentration, L-SC = leaf starch concentration, L-NSC = leaf non-structural carbohydrates, L-C = leaf total C, L-N = leaf total N, L-P = leaf total P, L-C:N = leaf C:N ratio, L-C:P = leaf C:P ratio, L-N:P = leaf N:P ratio, LB = leaf biomass, S-SSC = stem soluble sugar concentration, S-SC = stem starch concentration, S-NSC = stem non-structural carbohydrates, S-C = stem total C, S-N = stem total N, S-P = stem total P, S-C:N = stem C:N ratio, S-C:P = stem C:P ratio, S-N:P = stem N:P ratio, SB = stem biomass, R-SSC = root soluble sugar concentration, R-SC = root starch concentration, R-NSC = root non-structural carbohydrates, R-C = root total C, R-N = root total N, R-P = root total P, R-C:N = root C:N ratio, R-C:P = root C:P ratio, R-N:P = root N:P ratio, RB = root biomass.

Rhizosphere soil TP was significantly positively correlated with the SSC of different organs, but for roots, SSC was also correlated with the total nitrogen (TN), AN, and AP of rhizosphere soil. Except for TC, other measured soil factors were significantly negatively correlated with SC and were positively correlated with the biomass of different organs. A significant positive correlation was only found between root NSCs and rhizosphere soil TP, AN, AP (Fig. 5). The relationships between rhizosphere soil nutrients and plant stoichiometric characteristics differed among plant organs, but were significantly positively correlated with plant N, and negatively correlated with C:N. Rhizosphere soil nutrients were more strongly related to root stoichiometric characteristics than leaf and stem stoichiometry.

**Figure 6:**
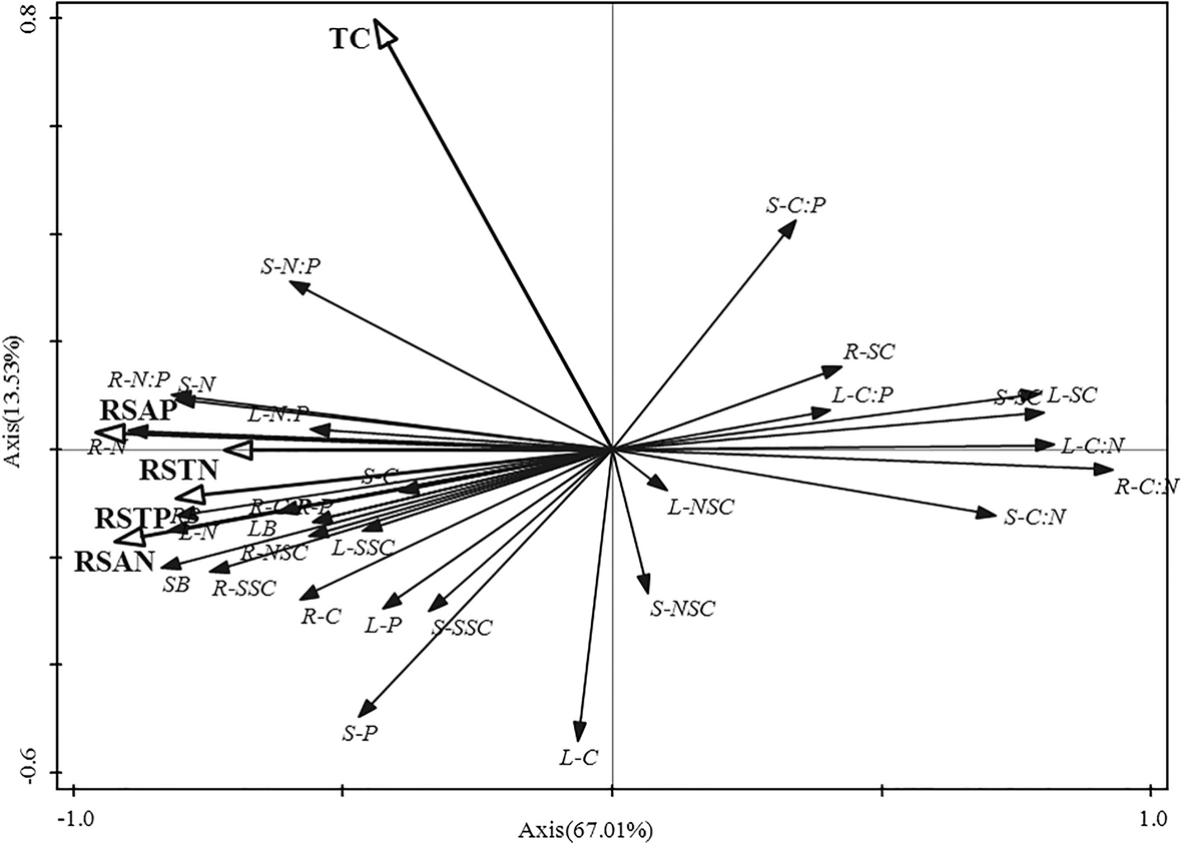
Redundancy analysis (RDA) between soil factors and ecological stoichiometry and NSCs of *R. soongorica*. RSAP = rhizosphere soil available P, TC = rhizosphere soil total carbon, RSTP = rhizosphere soil total phosphorus, RSTN = rhizosphere soil total nitrogen, RSAN = rhizosphere soil available N, L-SSC = leaf soluble sugar concentration, L-SC = leaf starch concentration, L-NSC = leaf non-structural carbohydrates, L-C = leaf total C, L-N = leaf total N, L-P = leaf total P, L-C:N = leaf C:N ratio, L-C:P = leaf C:P ratio, L-N:P = leaf N:P ratio, LB = leaf biomass, S-SSC = stem soluble sugar concentration, S-SC = stem starch concentration, S-NSC = stem non-structural carbohydrates, S-C = stem total C, S-N = stem total N, S-P = stem total P, S-C:N = stem C:N ratio, S-C:P = stem C:P ratio, S-N:P = stem N:P ratio, SB = stem biomass, R-SSC = root soluble sugar concentration, R-SC = root starch concentration, R-NSC = root non-structural carbohydrates, R-C = root total C, R-N = root total N, R-P = root total P, R-C:N = root C:N ratio, R-C:P = root C:P ratio, R-N:P = root N:P ratio, RB = root biomass.

RDA analyses showed that measured rhizosphere soil nutrients affected the stoichiometric characteristics and NSCs of *R. soongorica* (Fig. 6). The first axis explained 67.01% of the total variation and the second axis accounted for 13.25% of the total variation, and therefore 79.26% of the variation in plant stoichiometric characteristics and NSC traits were explained by soil factors. Among the five soil factors, rhizosphere soil AP was the most significant variable that affected the stoichiometric characteristics and NSCs of *R. soongorica* (*P* = 0.002), explaining 55.1% of the total variance. Rhizosphere soil AN and TP were the next most important variables, explaining 51.2% and 39.7% (*P* = 0.002) of the total variance, respectively. Rhizosphere soil AP, AN and TP were negatively correlated with the first axis, with correlation coefficients of 0.892, 0.887, 0.802, respectively (Fig. 6). Therefore, rhizosphere soil AP, AN and TP concentrations were important factors affecting the plant stoichiometric characteristics and NSCs of *R. soongorica*.

## Discussion

### Effects of N addition on plant biomass

Plant growth is closely related to nutrients, especially in nutrient-deficient soil. Soil nitrogen content is low in the arid and semi-arid regions of northwestern China, and is one of the main factors limiting plant productivity in the region^27^. Contrary to our first hypothesis, we found that N addition up to 9.2 g m^−2^ year^−1^ significantly increased the biomass and RGR of different organs, but biomass and RGR significantly decreased at highest level of N addition (13.8 g m^−2^ year^−1^). This result is consistent with the results of Jin et al.,^13^ and previous studies have found that the study area is N-limited^28^, explaining why plant growth was enhanced at 0–9.2 g m^−2^ year^−1^ N addition. This finding indicates that appropriate N addition is beneficial to the growth of *R. soongorica* in the Gobi desert region, and the N saturation value is between 9.2 and 13.7 g m^−2^ year^−1^. Once N addition exceeds the maximum N requirements for plant growth, plants will be less sensitive to N addition^29^.

A previous study has shown that plants can increase their allocation of photosynthetic products to belowground organs under lower 0.15 mM N conditions^30^, but biomass allocation patterns are species-specific^4,31^ and also vary with the amount of N addition. We found that the RGR of roots under N addition (up to 9.2 g m^−2^ year^−1^) was significantly higher than that of the shoot, thus resulting in a higher belowground : aboveground ratio. This result was inconsistent with the findings of Ai et al. who found that the rate of biomass increase of *Bothriochloa ischaemum* was more rapid aboveground than belowground^4^, also failing to support our first hypothesis. This discrepancy may be because *R. soongorica* allocated additional N from treatments to root biomass production over leaf and stem biomass production to better access water in the arid Gobi Desert region.

### Effects of N addition on NSCs

NSCs are the products of photosynthesis and provide energy for plant growth and metabolism, playing an important role in plant responses to environmental changes^32^. Some studies have reported that N addition increased NSC accumulation^31^, but other studies found that N addition had no effect on or even decreased NSC concentrations^4,9^. In this study, we found that NSC concentrations of different organs of *R. soongorica* significantly increased with mild to moderate N addition (0–9.2 g N m^−2^ year^−1^), but slightly decreased under the highest N addition treatment (13.8 g N m^−2^ year^−1^). This result failed to support our second hypothesis and was consistent with the results of other studies that found that low N addition contributes to the accumulation of soluble carbohydrates, thereby increasing their concentrations, but high N addition can significantly reduce concentrations^33^. Meanwhile, some studies have reported that the accumulation of higher NSC concentrations can balance the osmotic pressure of cells^34^ and be used to cope with environmental stress. Thus the high NSC concentrations in different organs under the moderate N addition treatment (9.2 g m^−2^ year^−1^) in our study indicate that adding proper amount of N will benefit for NSC accumulation and potentially improve the resistance of *R. soongorica* to water deficits in the Gobi Desert region, aiding the successful settlement of *R. soongorica* in this region.

We found that roots increased NSC concentrations more than leaves and stems under N addition, which is consistent with previous studies^4,31^. While we found that root NSC concentrations were higher than leaf and stem NSCs in our study, Ai et al^4^ found that aboveground NSC concentrations were higher than belowground NSC concentrations; this difference may be related to characteristics of C allocation and transport^35^. In order to facilitate water absorption and survive in the Gobi Desert, *R. soongorica* may have allocated more carbon to the roots than to leaves and stems^36^. Some studies have found that N addition significantly affected soil P concentrations, and soil P concentrations had significant effects on belowground starch and NSC concentrations, so N addition can accordingly have strong positive effects on belowground biomass NSC concentrations^4^. We found that N addition significantly increased rhizosphere soil AP, AN and TP concentrations, but the three major soil factors exerted different influences on the NSC concentrations of different organs of *R. soongorica*. The effects of soil nutrients on root NSC concentrations were significantly positive, but we observed no significant positive effects on leaf and stem NSC concentrations (Fig. 5), confirmed the conclusions of prior studies.

### Effect of N addition on plant stoichiometry

Stoichiometry reflected the utilization ability of C, N and P for plants, which are susceptible to environmental changes^37^. Many studies have concluded that N addition will result in higher soil N availability, and thus enhanced N concentrations and lower C:N ratios in many species^38,39^. Some studies also found that N addition significantly increased the C and N contents of plants and thus had no effect on C:N ratios^4,14^, but the stoichiometry of different organs can respond differently to environmental changes^40^. In this study, we found that the N_2_–N_3_ addition treatments significantly increased leaf and stem N concentrations and reduced leaf and stem C:N ratios, and all three N addition treatments significantly increased root N concentrations and reduced root C:N ratios in *R. soongorica*. This may be because available N in rhizosphere soil was significantly enhanced by N addition, but treatments had only minimal effects on total carbon. N addition also had greater effects on roots than on other organs, consistent with our third hypothesis and the results of Xiao et al.^31^, who reported that N addition increased N concentrations in roots compared to shoots. A plant’s N:P ratio has been interpreted as an indicator of N and/or P limitation^41,42^. It is widely accepted that N:P ratios < 10 indicate N limitation. *R. soongorica* displayed very low N:P ratios due to relatively high P concentrations in the higher N addition treatment, indicating that these plants remained N-limited despite the massive N doses that were applied. This result is consistent with the research of Wang et al.^43^, who found that the N:P ratio of *R. soongorica* was lower in the arid desert region, but is inconsistent with the results of Niu et al.^44^, who found that the N:P ratios of six shrubs (including *R. soongorica*) in the desert of the Alxa Plateau were all higher than 10. These inconsistent results may be due to variation among ecosystem types and environmental factors^45^.

## Conclusions

Total plant biomass and the biomass of different organs significantly increased with a range of N addition treatments (0–9.2 g m^−2^ year^−1^), and root biomass increased more rapidly than the leaf and stem biomass, resulting in higher belowground:aboveground ratios. N addition enhanced soluble sugar concentrations, but reduced the starch concentrations of all organs, and root NSC concentrations significantly increased under all N addition, but leaf and stem NSC concentrations only increased significantly under the N_1_ and N_2_ treatments. Plant N concentrations increased under higher N addition treatments (9.2–13.8 g m^−2^), but treatments had no significant effect on C and P concentrations, thus resulted in significantly reduced C:N ratios. N addition significantly increased the available N (AN), available P (AP) and total phosphorus (TP) of rhizosphere soil, and these soil nutrient variables were significantly positively correlated with root NSC concentrations. However, rhizosphere soil nutrients had stronger relationships with root stoichiometric characteristics than leaf and stem stoichiometric characteristics. This indicates that N addition alters the biomass, NSC concentration, N concentration, C:N ratio, and N:P ratio of different organs, though roots responded more strongly than stems or leaves, and rhizosphere soil AP, AN and TP concentrations were important factors affecting the NSC concentrations and stoichiometric characteristics of *R. soongorica*.

## Materials and methods

### Plant materials and growth conditions

*R. soongorica* is plentiful in the Gobi experimental fields at the Linze Inland River Basin Research Station (39°21′ N, 100°02′ E, 1400 m asl), Chinese Academy of Sciences. We harvested and dried fruiting branches of *R. soongorica* with mature seeds in October 2017. We beat the dried fruiting branches and collected the fallen seeds. After the seeds were soaked in water for 5–10 min, filled seeds would sink to the bottom, and we collected and air-dried these seeds, saving them in sealed plastic bags to nurture seedlings in the following year.

The experiment was also conducted at the Linze Inland River Basin Research Station. Average annual precipitation at the study site is 117 mm, and the mean annual temperature is 7.6 °C. Before the start of the experiment, soil samples (0–20 cm, 20–40 cm soil layer) without vegetation were collected at the Gobi experimental field in October 2017, and we removed visible plant residues, roots, stones and other materials, crushing the larger clods of soil. After air-drying, the soil samples were sieved through a 5 mm mesh and each soil layer was mixed. Initial soil chemical properties are presented in Table 2.

**Table 2:** Soil chemical properties in the pots before the begging of experiment.

Soil depth cm	Organic matter g kg^−1^	Total N g kg^−1^	Total P g kg^−1^	Available N mg kg^−1^	Available P m g kg^−1^	pH
0_–_20	1.48	0.4	0.73	26.9	189.9	7.6
20_–_40	0.85	0.29	1.48	16	123.8	7.7

### Experimental design

In March 2018, we added the collected soil samples to pots with a diameter of 34 cm and a height of 40 cm, first adding the 20–40 cm soil layer and then adding the 0–20 cm soil layer at the top. We sowed full seeds of *R. soongorica* into the pots, and soil water content was maintained at > 80% of field capacity during seedling establishment. After emergence, two seedlings with heights around 5 cm were retained in each pot for one year of growth. We initiated the experiment in May 2019, and one seedling was maintained in each pot after removal of the excess plant.

Nitrogen levels were designed according to nitrogen deposition levels in the desert region^18^, and four treatments received additional N in the form of urea at the rates of 0 (N_0_), 4.6 (N_1_), 9.2 (N_2_), and 13.8 (N_3_) g N m^−2^ year^−1^. Each treatment had six replicates. Urea [CO(NH _2_)_2_] has an N content of 46.7%, so the amount of urea applied was 0.8865, 1.7730, and 2.6595 g for treatments N_1_, N_2_, and N_3_ treatments, respectively. N was applied equally in the beginning of May, June, July, August, September and October in 2019, as a solution of urea in the same volume of deionised water, and the N_0_ treatment received the same volume of water at the same times.

### Sampling and chemical analysis

The six seedlings of *R. soongorica* for each treatment were sampled at the end of October. The whole seedlings of *R. soongorica* were taken out of the pots, the roots were wrapped with plastic wrap to prevent water loss, and all samples were taken back to the laboratory. We divided the seedlings into 3 parts: leaves, stems, and roots. Each part was washed with distilled water, and excess water at the surface was removed with blotting paper. We weighed all samples, placed them in labeled envelopes, and oven-dried each sample at 80 °C in an oven until they reached a constant weight to assess dry mass. Finally, the dried samples were ground using a Wiley Mill until they were sufficiently fine to pass through a 40-mesh sieve and stored for chemical analysis. Upon removal of each seedling from the pot, we collected the soil that was adhered to the root system using a brush and considered these samples rhizospheric soil. Each of these soil samples was air-dried and passed through a 2-mm-mesh sieve for chemical analysis.

We calculated NSC concentrations by summing soluble sugar concentrations and starch concentrations^4^, and the concentrations of soluble sugars and starch were determined using an anthrone method^46^. Total N concentrations were measured using the Kjeldahl method. Total P concentrations were determined using the molybdenum blue colorimetric method. Total C concentrations were determined using the dichromate oxidation method. Available N was measured using the alkaline diffusion method, and available P was measured using the sodium bicarbonate leaching-molybdenum antimony anti-colorimetric method^47^.

### Data analysis

The relative growth rate (RGR, g d^−1^) was calculated as follows:

$$RGR = \frac{{\ln W_{2} - \ln W_{1} }}{t}$$

where W_1_ is the initial biomass, W_2_ is the biomass under the N addition treatment (N_0_–N_3_), and t is the time interval between the two measurements.

We used one-way analysis of variance (ANOVA) to test the effects of nitrogen on biomass, NSC concentrations, C, N and P stoichiometry of different organs. Duncan’s multiple range tests were used for multiple comparisons among each treatment. Data analyses were performed using SPSS Statistics procedure (Version 13.0) and figures were drawn using Origin 8.

### Research involving plants

Experimental research and field studies on *Reaumuria soongorica* plants complied with relevant institutional guidelines.

## Supplementary Information

Supplementary Information.

## Data Availability

All datasets generated and/or analyzed during the current study are included in this article.
